# Research Progress and Translational Perspectives of Piezoelectric Materials in Dental Implant Surface Engineering

**DOI:** 10.3390/jfb17060278

**Published:** 2026-06-04

**Authors:** Xu Cao, Jiangqi Hu, Qian Pang, Qingsong Jiang, Su Chen, Bin Luo

**Affiliations:** Beijing Stomatological Hospital, Capital Medical University, Beijing 100070, China

**Keywords:** piezoelectric materials, dental implants, surface engineering, piezocatalysis, osteoimmunomodulation, clinical translation

## Abstract

The long-term stability of dental implants is limited by multiple factors, including peri-implant infection, impaired osseointegration, and poor soft tissue sealing. Compared with conventional passive surface modification strategies, piezoelectric materials can convert mechanical energy into local electrical signals under occlusal loading, cell traction, or ultrasonic stimulation. With the aid of defect engineering, heterostructure construction, and co-catalytic design, these materials can also induce the generation of reactive oxygen species and reactive nitrogen species, thereby enabling on-demand antibacterial activity. This review systematically summarizes the bioelectric basis of bone tissue and clarifies how piezoelectricity and piezocatalysis may be used in dental implant surface engineering. Their applications are discussed in terms of antibiofilm and antibacterial activity, osteogenesis and osseointegration, osteoimmunomodulation, soft tissue healing, and temporally programmed therapy. In addition, this review also discusses issues that remain unresolved, such as polymer-based composite systems, realistic activation windows, evaluation standards, device–material integration, and multi-omics validation. Overall, piezoelectric surface engineering is evolving from a single osteogenesis-oriented strategy into an integrated platform that coordinates infection control, immune remodeling, and osseointegration. However, the actual effectiveness of its clinical application still needs to be determined through more rigorous mechanism analysis, long-term stability assessment, biosafety assessment, and standardized preclinical research.

## 1. Introduction

With the advancement of implant technology, dental implants have become one of the preferred methods for restoring missing teeth. Currently, the main materials used for dental implants include pure titanium, titanium alloys, and zirconia-based ceramics. Titanium and titanium alloys remain the primary choice because of their good mechanical strength, corrosion resistance, and biocompatibility [1,2,3]. Zirconia implants have also attracted attention due to their tooth-like color, low plaque accumulation, and metal-free nature. However, their brittleness, aging behavior, and relatively limited flexibility for surface modification still restrict their wider use in some clinical situations [4,5,6]. The long-term success of an implant depends not only on the mechanical properties of the material but also on its biological quality—specifically, the integration with both soft and hard tissues. Key factors include infection control, the quality of osseointegration, and the efficacy of the soft tissue barrier [7,8,9].

**Figure 1:**
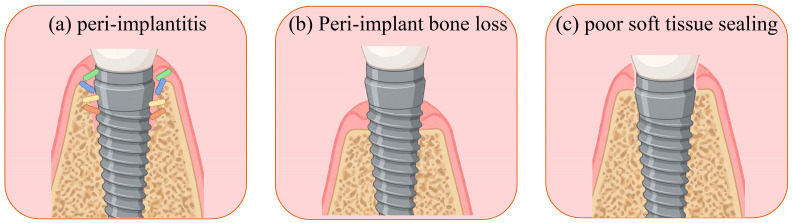
Major biological challenges of the peri-implant interface. (**a**) Peri-implantitis. (**b**) Peri-implant bone loss. (**c**) Poor soft tissue sealing.

Although dental implants have a high survival rate, the peri-implant interface still faces three major biological challenges (Figure 1). Figure 1a shows peri-implantitis, in which bacterial biofilms and inflammation develop around the implant. Figure 1b shows peri-implant bone loss and impaired osseointegration, which compromise implant stability. Figure 1c shows poor soft-tissue sealing, which weakens the mucosal barrier and promotes bacterial invasion. These problems remain major causes of functional impairment, secondary intervention, and even implant failure [10,11,12]. Traditional surface modification strategies—such as surface roughening, hydrophilization, hydroxyapatite coating, and the incorporation of metal ions or antimicrobial peptides—can enhance protein adsorption, cell adhesion, and early-stage osteogenesis. However, most of these methods still rely on static or passive regulation; they often fail to simultaneously achieve both anti-infection and pro-regeneration effects at the same interface, and they lack the dynamic outputs corresponding to the distinct stages of tissue healing [2,13,14].

Piezoelectric materials are receiving increasing attention mainly because of their “self-powered” properties. Since Fukada and Yasuda first demonstrated the piezoelectric effect in bone tissue [15], extensive research has indicated that collagen-associated piezoelectric effects, mechanically induced streaming potentials, and endogenous bioelectrical signals generated under conditions of injury or stress collectively constitute a critical electrophysiological microenvironment within bone that participates in regulating osteocyte migration, differentiation, and bone remodeling [16,17,18,19,20]. Therefore, constructing a piezoelectric or electroactive layer on the implant surface that can respond to occlusal forces, cell traction, or ultrasonic (US) stimulation may provide a favorable local electrical environment for the implant interface without the need for an external power source [21,22,23,24].

Piezoelectric surfaces are not traditional electrically stimulating surfaces. Through appropriate band structures, polarization states, and interfacial designs, mechanical stimulation can not only induce charge separation but also drive interfacial redox reactions. These reactions can generate reactive oxygen species (ROS) and reactive nitrogen species (RNS), thereby achieving on-demand antibiofilm and antibacterial activity [25,26,27,28,29]. Therefore, piezoelectric surfaces have the dual potential to promote tissue repair and control infection. This characteristic is particularly important for dental implants, as they are continuously exposed to the complex microbial communities and periodic mechanical loads present in the oral environment.

This review is structured around five interconnected dimensions: clinical challenges, bone bioelectricity, material systems, functional mechanisms, and translational assessment. First, key clinical issues are explored, including peri-implant infections, impaired osseointegration, and poor soft tissue sealing. Second, the natural electrical phenomena inherent in bone tissue are discussed. Third, BaTiO_3_ (BTO), SrTiO_3_/BST, CaTiO_3_, Bi_2_WO_6_/BFBT, and related electroactive synergistic surfaces are categorized and summarized. Fourth, focuses on four major functions: antibiofilm and antibacterial activity, osteogenesis and osseointegration, osteoimmunomodulation, and soft tissue healing. Finally, a standardized framework of evidence is compiled to evaluate the feasibility of applying these strategies to dental implants. This review aims to define surface design principles that are truly applicable to clinical settings.

## 2. Bioelectricity of Bone Tissue and Theoretical Basis of Piezoelectric Surfaces

### 2.1. Bioelectric Basis of Bone Tissue

Bone is a tissue possessing electrical properties. Classic studies have demonstrated that the non-centrosymmetric structure of Type I collagen molecules generates a piezoelectric response under mechanical loading. Simultaneously, fluid flow within the canalicular-lacunar system induces streaming potentials. These phenomena constitute the basis of mechano-electrical coupling in bone tissue [15,16,17]. Relatively lower potential values are observed in regions under compression, while relatively higher potential values are found in regions under tension; this phenomenon provides a crucial foundation for understanding the relationship between bone remodeling and stress distribution. However, this phenomenon is also influenced by hydration status, tissue anisotropy, and the surrounding fluid environment [16,17].

The electrical microenvironment of bone tissue has four major sources: collagen-related piezoelectricity, fluid flow-associated potentials, endogenous electric fields formed after injury, and local potential changes induced by external electrical stimulation at the bone–material interface [16,17,18,30,31]. These electrical signals enable bone cells to sense mechanical stimulation and initiate the repair process by regulating cell membrane potential, ion channels, and the cytoskeleton.

In osteogenesis, moderate local electrical stimulation can alter the polarization state of the cell membrane and promote calcium ions (Ca^2+^) influx, thereby activating signaling pathways associated with osteogenic differentiation, including the MAPK/ERK, PI3K-AKT, and Wnt/β-catenin pathways [18,30,31].

In immune regulation, macrophages are among the first cells to be recruited to the implantation site. Their dynamic activation state exerts a significant influence on inflammation, angiogenesis, and bone formation [13,32,33,34,35,36]. Piezoelectric surfaces not only have a direct stimulating effect on osteoblasts, but also affect the interaction between immunity and osteogenicity, thereby regulating the quality of bone repair. Local piezoelectric potentials, controlled ROS/RNS levels, ionic signals, and nanotopographical cues can regulate macrophage activation and cytokine secretion [35]. During the early inflammatory or infection-control stage, a transient M1 response may be beneficial by enhancing phagocytosis, bacterial clearance, and the secretion of inflammatory mediators, such as TNF-α, IL-1β, and iNOS. However, excessive or prolonged M1 activation may aggravate inflammation and impair tissue repair. Therefore, a timely shift toward an M2 reparative phenotype is critical for successful bone healing [32,33,36].

**Figure 2:**
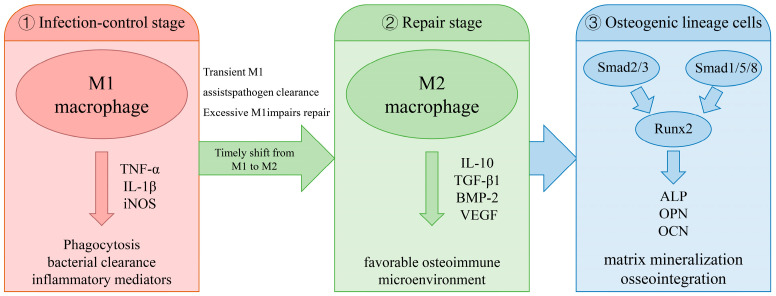
Immune–osteogenic interaction regulated by piezoelectric bioelectrical cues.

During the repair stage, M2 macrophages can establish a favorable osteoimmune microenvironment by secreting anti-inflammatory and pro-regenerative mediators, such as IL-10, TGF-β1, BMP-2, and VEGF [13,32]. These paracrine signals further act on peri-implant bone marrow mesenchymal stem cells (BMSCs) and osteogenic lineage cells. They activate osteogenic signaling pathways, including Smad2/3 and Smad1/5/8, and promote Runx2-mediated osteogenic differentiation [37,38]. Therefore, the biological effects of piezoelectric surfaces should not be simply interpreted as direct electrical stimulation of osteoblasts. Instead, they should be viewed as a coordinated process involving bioelectric cues, stage-specific immune regulation, macrophage–BMSC/osteogenic cell interactions, angiogenesis, matrix mineralization, and early osseointegration (Figure 2).

### 2.2. Piezoelectric Effect and Piezocatalysis

The essence of the piezoelectric effect is a change in polarization potential induced by mechanical deformation. For implant surfaces, the material’s ability to convert weak mechanical inputs into biologically significant electrical outputs depends on multiple factors, including crystal structure and phase composition, polarization state, orientation and thickness, structural deformability, interfacial band alignment, and the amplitude and frequency of mechanical stress [21,22,25,39,40].

When piezoelectric surfaces are exposed to cyclic mechanical stimulation, especially ultrasound or low-intensity pulsed ultrasound (LIPUS), spatially separated positive and negative polarization charges are generated at the interface. These charges have different functional roles. Negatively polarized regions may attract cations, such as Ca^2+^, and promote local apatite nucleation. In contrast, positively polarized regions may attract anionic groups and participate in oxidation reactions. For example, barium strontium titanate (BST) nanorod-array coatings show load-dependent surface potential distributions. Under downward loading, the rod tops become negatively polarized, while the lower regions gradually shift toward a positive potential. Under lateral loading, the positive and negative potential patterns change with the bending angle of the rods and the loading direction [41].

During piezocatalysis, these opposite polarization regions promote the separation of electrons and holes. Electrons at negatively polarized or electron-rich sites can reduce O_2_ to ·O_2_^−^ and contribute to H_2_O_2_ formation. In contrast, holes or positively charged regions can oxidize H_2_O/OH^−^ to ·OH. Representative systems, such as piezoTi, Al-SrTiO_3_/TiO_2_ nanotubes (Al-STNT), and (BiFe)_0_._9_(BaTi)_0_._1_O_3−x_ (BFBT), show that improved charge transfer, transient current responses, or more suitable redox potential levels can enhance ROS generation under ultrasound stimulation [42,43,44]. Therefore, alternating positive and negative charges should be understood as dynamic polarization fields. These fields regulate both ion adsorption/mineralization and piezocatalytic redox reactions. However, the ultrasonic environment does not provide mechanical compression alone. Cavitation, microjets, local heating, and general sonochemical effects may also contribute to the final biological outcomes. Therefore, in studies of acoustically activated implant surfaces, it is difficult to strictly attribute antibacterial or pro-regenerative effects to the piezoelectric mechanism itself if depolarized controls, non-piezoelectric chemical analogues, or temperature and cavitation monitoring are absent [27,28,45]. This remains one of the most common and important weaknesses in the current literature.

### 2.3. Effective Mechanical Inputs in Dental Implant Applications

For dental implants, mechanical input is not a single-variable factor. During clinical service, at least three potential activation sources may be involved. The first is the macroscopic cyclic load generated by mastication. The second is the microscale mechanical force produced by the adhesion and traction of osteocytes, fibroblasts, and macrophages. The third is exogenous mechanical waves, such as LIPUS or therapeutic US. These inputs differ substantially in amplitude, frequency, duration, and spatial distribution. As a result, the same surface may generate markedly different electrical and catalytic outputs under different conditions [23,31,46,47,48,49].

Mastication can provide a large macroscopic load. However, the transfer of occlusal force to a thin coating is indirect and highly non-uniform. The maximum human bite force varies widely, with reported values ranging from approximately 50 to 900 N. This variation depends on individual conditions, tooth position, prosthesis design, and measurement method [50]. However, only a small fraction of this load is converted into local strain on the implant surface. Excessive micromotion is also undesirable. Interfacial micromotion has been associated with impaired osseointegration and fibrous tissue formation. Although a universal threshold remains debated, values of 50–150 μm are commonly discussed in implant studies [51,52]. Therefore, mastication should mainly be considered as a possible long-term source of low-level electrical stimulation, rather than a reliable trigger for strong piezocatalytic activity.

Cell traction is much weaker and acts over a more localized area. Osteoblasts, fibroblasts, macrophages, and epithelial cells generate traction forces through focal adhesions at the nano- to microscale. For example, osteoblast traction stress near the leading edge has been measured at approximately 0.22–0.25 nN/μm^2^ [53]. These forces may be sufficient to regulate local surface potential, focal adhesion maturation, cytoskeletal organization, and mechantransduction pathways. However, they are unlikely to generate a uniform or high-intensity catalytic output over the entire implant surface. Therefore, the piezoelectric response induced by cell traction should be interpreted as a local mechanobiological signal, rather than an independent antibacterial activation mechanism.

Based on current evidence, US remains the most controllable external stimulus. The piezoTi surface reported by Li et al., the Al-STNT system constructed by Pan et al., and the BaTiO_3−x_/LA platform developed by Sun et al. all achieved significant antibacterial effects within an activation window of approximately 1.0 MHz, 1.5 W/cm^2^, 50% duty cycle, and 5–15 min of treatment [42,43,54]. Wu et al. further shifted attention toward LIPUS by developing BST arrays, with the aim of lowering the threshold for clinical application [41]. However, energy coupling in the real oral environment is also affected by soft tissue thickness, probe angle, coupling medium, implant position, and bone density. Ultrasound attenuation in soft tissue increases with frequency and is usually expressed as dB/cm/MHz [55].Therefore, if studies fail to incorporate the energy transfer among US, tissue, and implant into their evaluation systems, the effective parameters identified in the laboratory will be difficult to translate into clinical practice [45,47,56,57,58].

Long-term stability in the oral environment is another key requirement. Piezoelectric coatings are exposed to cyclic occlusal loading, saliva, pH fluctuations, proteins, acquired pellicles, polymicrobial biofilms, thermal cycling, cleaning procedures, and repeated activation. These factors may change coating adhesion, polarization retention, defect stability, ion release, catalytic activity, and the generation of wear particles. Therefore, biologically meaningful outputs should be confirmed after aging under oral-like conditions. They should not be inferred only from short-term piezoresponse force microscopy (PFM) measurements or single-cycle ROS assays.

### 2.4. Mechanistic Classification of Piezoelectric Surface Functions

From a materials science perspective, the biological outputs of piezoelectric surfaces can generally be divided into three categories. The first category is a mild output dominated by electrical signals. For example, the time-dependent potential changes on polarized CaTiO_3_ surfaces mainly regulate macrophage polarization and osteogenic pathways. The second category is a reactive output dominated by piezocatalysis or piezo-sonocatalysis. In this case, mechanical activation drives electron–hole separation and induces the generation of ROS or RNS. The third category is a composite output involving heterojunctions, co-catalysis, charge storage, or ion release. Its final biological phenotype is often the combined result of electrical stimulation, catalytic reactions, ionic signaling, and topographical regulation [43,44,54,59,60,61,62,63,64].

Therefore, the identification of a piezoelectric surface should not rely solely on PFM phase switching or surface potential mapping at a single time point. Instead, several aspects should be considered together: whether the material shows reproducible evidence of mechano-electrical conversion; whether it can generate electrical parameters under realistic stimulation conditions that are consistent with the cellular or bacterial outcomes; and whether its antibacterial or osteogenic effects can be distinguished from those of depolarized controls or non-piezoelectric controls. Only when these three levels of evidence are established can the proposed piezoelectric mechanism be considered convincing [27,28,46].

## 3. Construction Strategies for Representative Piezoelectric Material

With the increasing interest in piezoelectric surfaces for dental implants, research in this field has shown several clear trends. Material systems have become more diverse, structural designs have shifted toward micro- and nanoscale architectures, and the functional goal has shifted from single osteogenic enhancement to coordinated antibacterial–immune–osseointegration regulation. Table 1 summarizes the most representative piezoelectric surface systems currently investigated for implant applications (Table 1).

### 3.1. BaTiO_3_-Based Surfaces

BTO is currently one of the most representative lead-free perovskite piezoelectric materials in dental implant surface research. Its advantages lie not only in its well-established piezoelectric properties, but also in the relatively mature surface fabrication routes. Typically, a titanate precursor layer is first formed on the titanium surface through alkaline hydrothermal pretreatment. This layer is then hydrothermally converted in a Ba^2+^-containing system, allowing nanorod- or nanoparticle-like BTO layers to be grown in situ on the substrate [25,42,54,65,66]. This substrate-derived coating strategy is naturally favorable for interfacial bonding.

Li et al. constructed BTO nanostructures on Ti surfaces and further deposited Au nanoparticles as cocatalysts, thereby obtaining the piezoTi surface. Under ultrasonic stimulation, Au acted as an electron sink and markedly enhanced charge carrier separation. The material not only directly killed bacteria and downregulated genes associated with *S. aureus* biofilm formation, but also enhanced macrophage phagocytic activity. In an osteomyelitis model, it achieved both anti-infective effects and improved osseointegration [42]. This work marks a shift in BTO surface research from simple osteogenic promotion toward coordinated antibacterial, immunomodulatory, and bone-regenerative functions.

Xu et al. proposed the concept of surface-confined piezocatalysis. BTO@Au particles were semi-embedded in a polymer surface, restricting electron release and oxidative stress mainly to the narrow implant–bacteria interface. This strategy suggests that infection control does not necessarily depend on a higher total amount of ROS. The key issue is whether the reaction occurs at the interface where it is most needed. This concept was later extended to an ex vivo human root canal reinfection model, indicating that narrow anatomical spaces in the oral cavity may represent one of the most advantageous application scenarios for piezoelectric surfaces [67].

Sun et al. introduced oxygen vacancies into BTO nanorods and covalently grafted L-arginine to construct a BaTiO_3−x_/LA hybrid coating. Under ultrasonic stimulation, ROS triggered NO release. NO then rapidly reacted with ·O_2_^−^ to generate ONOO^−^, forming a ROS–NO–RNS cascade amplification system. In a rat tibial Methicillin-resistant Staphylococcus aureus (MRSA) infection model, this system achieved an in vivo antibacterial rate of 97.54% after ultrasonic activation and reached a bone volume fraction (BV/TV) of 34.26% at 8 weeks [54]. These results indicate that it possesses the potential to promote bone repair while simultaneously clearing infection. This study also suggests that defect engineering does not simply follow the “more is better” principle. Although oxygen vacancies can enhance catalytic activity, they may also weaken some piezoelectric advantages of pristine BTO. Therefore, a balance must be achieved between catalytic enhancement and polarization stability.

### 3.2. Strontium-Containing Titanates and BST Systems

Strontium-containing titanates have attracted considerable attention for two main reasons. First, Sr^2+^ has well-documented effects in promoting osteogenesis, inhibiting bone resorption, and regulating immune responses. Second, nanoscale strontium titanate (SrTiO_3_) and its solid solutions or defect-engineered structures can exhibit electrical or piezoelectric-like behaviors under external stimulation [43,68,69]. SrTiO_3_ at room temperature has a centrosymmetric structure, the piezoelectricity reported in implant surface usually arises from nanoscale distortion, doping, oxygen vacancies, or heterointerfaces.

Pan et al. constructed Al-SrTiO_3_/TiO_2_ nanotube arrays on Ti surfaces using an anodized TiO_2_ nanotube template, followed by hydrothermal growth of SrTiO_3_ and Al doping. Al doping introduced oxygen vacancies and lattice distortion, thereby enhancing ROS generation under ultrasonic stimulation. In addition, this study used oral-related bacteria such as Porphyromonas gingivalis and Fusobacterium nucleatum, and evaluated antibacterial activity and osseointegration in a rat dental implant model. These designs substantially improved the relevance of the study to oral implant scenarios [43].

The SrTiO_3_ nanotube-based pneumatic nanogun platform reported by Wang et al. is closer to a responsive drug–device integrated surface. In this system, SrTiO_3_ nanotubes were used as carriers and loaded with polydopamine (PDA), NH_4_HCO_3_, and antibiotics. Near-infrared (NIR) irradiation induced gas release, enabling on-demand drug ejection. At the same time, sustained Sr^2+^ release promoted osseointegration. Although this system is not a typical piezoelectric sonocatalytic surface, its antibacterial efficiency of over 99% and Sr^2+^ release lasting more than 28 days indicate that titanate nanotube-based surfaces are not limited to piezoelectric functions alone. They can also serve as drug carriers and support temporally programmed therapy [60].

The BST nanorod arrays designed by Wu et al. reduced the threshold for effective response from both compositional and geometrical perspectives. Under LIPUS, this coating generated positively and negatively distributed surface potentials. It also promoted apatite deposition and osteoblast adhesion and proliferation. These findings suggest that coordinated regulation of the Ba/Sr ratio and surface morphology may allow implant surfaces to better adapt to clinically acceptable low-energy mechanical stimulation conditions [41].

### 3.3. Polarized CaTiO_3_ Surfaces

Compared with BTO or strontium-containing systems, CaTiO_3_ (CT) garners attention not because of a higher piezoelectric coefficient, but because its composition is compatible with the bone mineralization microenvironment and it exhibits stable surface electroactivity after polarization. The polarized CaTiO_3_ coating (CT-P) developed by Dai et al. showed a time-dependent electrical output even without ultrasonic stimulation. This output could regulate macrophage polarization and promote osteogenesis. When antibacterial activity was required, ROS generation could be activated by ultrasound, enabling a functional switch from repair promotion under normal conditions to antibacterial action [59]. The value of this type of study lies in its clear demonstration of a temporal correspondence between surface functions and different stages of tissue repair.

### 3.4. Bi_2_WO_6_ and Heterojunction Systems

Layered oxides such as Bi_2_WO_6_ have expanded the research focus from classical titanates to piezoelectric heterojunctions. Fan et al. grew Bi_2_WO_6_ nanocrystals on TiO_2_ nanowires to construct a TiO_2_/Bi_2_WO_6_ piezoelectric heterojunction. With the assistance of oxygen vacancies and heterointerfaces, this system achieved synergistic photothermal/photodynamic antibacterial effects under NIR irradiation. During the repair stage, it could generate local electrical signals through cell traction, thereby activating PI3K-AKT-related osteogenic pathways [61]. Although its antibacterial activation mainly relies on NIR, this study shows that heterojunction design is not merely intended to increase ROS production. More importantly, it can assign the antibacterial window and the bone repair window to different stimulation conditions.

### 3.5. Electroactive Synergistic Surfaces

The design of dental implant surfaces is gradually shifting from single-function piezoelectric coatings to electroactive platforms with synergistic functions. In these systems, biological performance depends not only on piezoelectric polarization, but also on charge separation, charge retention, interfacial redox reactions, ion release, drug delivery, and immunomodulation.

The TiO_2_-SnO_2_-RuO_2_ multilayer heterostructure constructed by Zhou et al. is closer to an electroresponsive pseudocapacitive energy-storage system than to a classical piezoelectric platform. Nevertheless, its built-in electric field and post-charging mode significantly enhanced surface antibacterial activity and improved osseointegration. This finding indicates that electrical output and charge carrier utilization efficiency are also critical dimensions in implant surface design [62].

Although the tannic acid–strontium (TA-Sr) metal–phenolic network coating reported by Liu et al. is not a piezoelectric surface, it provides an important reference for an immune-first surface strategy through early M2-biased macrophage polarization, BMSC recruitment, and promotion of osteogenic differentiation [70]. Therefore, it is included as a biologically informative model that integrates immunomodulation with osteogenesis-oriented surface design.

The PiezoTi surface, based on BaTiO_3_ nanostructures and Au cocatalysts, combines ultrasound-triggered ROS generation with macrophage-mediated antibacterial activity [42]. The BaTiO_3−x_/L-arginine coating further integrates oxygen-defect-enhanced piezocatalysis, NO-mediated radical chain reactions, and stage-specific immune regulation. This design improves the clearance of MRSA and promotes subsequent osteogenesis [54]. Polarized CaTiO_3_ coatings represent another temporal strategy. In this system, baseline electrical stimulation supports immunomodulation and osteogenesis, while ultrasound-triggered ROS generation provides on-demand antibacterial activity [59]. Similar synergistic concepts have also been reported in Al-doped SrTiO_3_/TiO_2_ nanotubes, defect-rich piezoelectric nanoreactors, TiO_2_/Bi_2_WO_6_ heterojunctions, and SrTiO_3_ nanotube-based drug delivery coatings [43,44,60,61].

Overall, clinically competitive dental implant surfaces are unlikely to rely only on simple piezoelectric single-function systems. Instead, they may develop into stage-adaptive composite platforms. These platforms should coordinate early infection control, immune remodeling, soft-tissue protection, and subsequent osseointegration within a clinically acceptable activation window.

### 3.6. Surface Construction Routes and Key Processing Considerations 

In situ hydrothermal growth remains the mainstream strategy for fabricating oxide-based piezoelectric surfaces. This approach usually begins with the formation of a titanate precursor layer on the titanium surface. The precursor is then converted into BTO, BST, or CT nanorods/nanowires under relatively mild conditions. The advantages of this method include controllable morphology, strong interfacial bonding, and good conformality on complex three-dimensional surfaces. However, subsequent heat treatment may introduce brittleness and residual stress. In addition, the formation of the piezoelectric phase and the polarization state cannot always be fully controlled [41,42,59,65,71].

The combination of anodization and hydrothermal treatment is particularly suitable for nanotube-based systems. Ordered TiO_2_ nanotubes can serve as mechanically interlocking templates. They also facilitate subsequent SrTiO_3_ formation, doping, and drug loading. Therefore, they play a key role in Al-STNT and SrTiO_3_ nanocannon systems [43,60,72]. However, multistep fabrication also increases the risk of batch-to-batch variation. Problems such as nanotube wall collapse, incomplete phase transformation, and local thickness heterogeneity need careful evaluation during scale-up manufacturing.

For many ceramic surfaces, polarization is a key factor that determines the upper limit of functional performance. Corona poling or high-voltage poling can markedly enhance surface potential and charge retention. However, the effects of long-term depolarization, sterilization, insertion torque, and cyclic oral loading on polarization stability remain insufficiently studied [41,59].

In addition, enhancement units such as oxygen vacancies, aliovalent doping, Au/semiconductor heterojunctions, and pseudocapacitive layers have evolved from additional optimization strategies into core variables in surface design. These units can simultaneously regulate band structure, charge carrier separation, ROS yield, and ion release. At the same time, they may increase structural complexity, make mechanistic attribution more difficult, and reduce manufacturing stability [42,43,44,54,61,62,73] (Table 2).

### 3.7. Polymeric and Organic–Inorganic Composite Piezoelectric Systems

Compared with inorganic ceramics, polyvinylidene fluoride (PVDF), poly(vinylidene fluoride-co-trifluoroethylene) (P(VDF-TrFE)), and their composites with silk fibroin, hydroxyapatite, BTO nanoparticles, and other components offer several advantages. These systems are flexible, processable, and suitable for fabricating large-area films or hydrogels. Recent studies have shown that such materials hold promise for bone defect repair, infected wound healing, and osteoimmune remodeling. For example, ultrasound-activated Silk-PVDF hydrogels can reshape the osteoimmune microenvironment through NRF2-related pathways. Broader reviews have also highlighted the potential of polymeric piezoelectric materials in craniomaxillofacial regeneration and dental therapy [20,57,74,75,76,77,78,79].

However, there is still a clear gap between polymeric piezoelectric systems and load-bearing dental implant surfaces. First, the long-term adhesion, wear resistance, and sterilization stability of flexible films on metallic threaded implants remain unclear. Second, the piezoelectric output of polymeric systems is usually highly dependent on molecular orientation, stretching, and polarization state. This makes process stability more complex than that of inorganic ceramics. Third, most current evidence comes from bone defect membranes, wound dressings, or scaffolds. Therefore, these systems may be more suitable as transmucosal barrier layers, local drug or ion carriers, or flexible synergistic components. For the intraosseous segment, inorganic or inorganic-dominant composite surfaces remain more realistic in the near term [20,57,74,75,76,77,78,80,81,82].

Poly(lactic acid) and poly(L-lactic acid) (PLA/PLLA), peptide assemblies, silk fibroin, collagen, and other biodegradable piezoelectric materials are also receiving increasing attention [83,84,85]. These materials can provide temporary mechanoelectrical signals. Their main value is not as long-term load-bearing coatings on implants. Instead, they may serve as temporary functional layers that deliver mechanoelectrical cues during a specific healing window and then degrade after completing their role [84,85,86,87].

Such systems may be particularly suitable for soft-tissue interfaces, guided bone regeneration or periodontal barrier membranes, neural/periodontal interfaces, local drug delivery, and early osteoimmune regulation. However, their use in dental implant systems is still at an exploratory stage. Key challenges include matching degradation kinetics with the timeline of osseointegration, maintaining piezoelectric output during degradation, avoiding acidic or pro-inflammatory degradation products, preventing particle release, and ensuring sterilization compatibility and coating adhesion on complex implant geometries.

### 3.8. III-Nitride Piezoelectric Semiconductors: Aluminum Nitride (AlN) and Gallium Nitride (GaN)

AlN and GaN have a wurtzite crystal structure, intrinsic piezoelectric polarization, good chemical stability, and compatibility with thin-film microfabrication processes. Spontaneous polarization and piezoelectric polarization in III–V nitrides have been well established [88,89]. In the biomedical field, GaN has shown good cytocompatibility before and after peptide functionalization. Recent studies further suggest that GaN-based surfaces may influence neuronal cell behavior through their surface properties or persistent photoconductivity [90,91]. AlN and AlGaN/GaN systems have also been explored as biocompatible semiconductor materials for cell-interface applications. However, direct evidence for the use of AlN/GaN coatings in dental implant surface engineering remains very limited.

### 3.9. Comparative Analysis of Representative Piezoelectric and Electroactive Strategies 

Although many piezoelectric or electroactive implant surfaces show similar biological effects, their activation conditions and main mechanisms of action differ greatly. Therefore, these strategies should not be evaluated only by their final antibacterial or osteogenic outcomes. A more meaningful comparison should consider whether the biological output is mainly driven by mild electrical stimulation, ultrasound-triggered piezocatalysis, defect- or heterojunction-enhanced charge separation, ion/drug release, stage-specific immune regulation, or a combination of these mechanisms.

Endogenous force-responsive surfaces have the advantage of not requiring external devices. However, their output intensity may be weak and spatially heterogeneous [28,59]. Ultrasound-activated systems can provide stronger and more controllable outputs, especially for the generation of reactive oxygen/nitrogen species. However, their effects depend on tissue attenuation, coupling conditions, and the safety window [42,67]. Defect engineering, doping, and heterostructure construction can improve charge separation and catalytic efficiency. However, they also make mechanistic attribution more difficult, because piezoelectric, sonochemical, ionic, photothermal, and topographical effects may overlap [61]. Electroresponsive pseudocapacitive surfaces and ion/drug release platforms further expand the design space. However, their charging protocols, release kinetics, fatigue resistance, and manufacturing reproducibility require more rigorous validation [62].

Overall, no single strategy is absolutely superior. Weak electrical outputs may be more suitable for promoting osteogenesis and immune regulation, whereas stronger piezocatalytic outputs may be more appropriate for short-term infection control. For clinical application, the most promising direction may be stage-designed surfaces that coordinate early antibacterial activity, immune remodeling, soft-tissue protection, and long-term osseointegration within a safe and reproducible activation window (Table 3).

## 4. Biological Functions of Piezoelectric Surfaces

**Figure 3:**
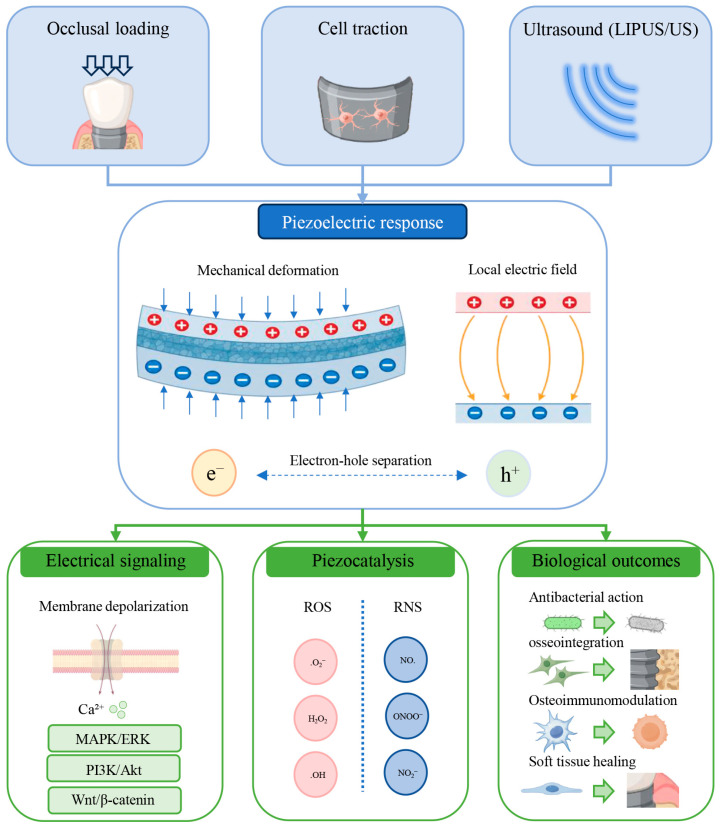
Piezoelectric stimulation-mediated biological regulation of implant surfaces.

The functional outputs of piezoelectric implant surfaces mainly fall into three interconnected directions: antibiofilm and antibacterial activity, osteogenesis and osseointegration, and osteoimmunomodulation. Figure 3 summarizes the main biological functions and interactions of piezoelectric implant surfaces. Under mastication, cell traction, or ultrasound stimulation, these surfaces may generate local piezopotentials, promote charge separation, induce ROS/RNS production, release ionic cues, and provide nanotopographical stimulation. These outputs are associated with antibacterial activity, osteogenic differentiation, osteoimmunomodulation, angiogenesis, and soft-tissue responses. Importantly, these functions are stage dependent. Strong reactive outputs may be more suitable for early infection control, whereas mild and sustained bioelectrical cues may better support immune remodeling, osteogenesis, and tissue repair.

### 4.1. Antibiofilm and Antibacterial Activity

Antibacterial activity is often the most rapidly detectable outcome of piezoelectric surface activation, which partly explains why this direction has expanded quickly in recent years. Dynamic mechanical stimulation, such as US, can induce local positive and negative charges on piezoelectric surfaces and drive redox reactions. This process leads to the generation of reactive species such as ·O_2_^−^, ·OH, and H_2_O_2_. In some cascade systems, stronger nitrosative stress involving NO and peroxynitrite (ONOO^−^) can also be introduced [27,42,43,44,54,67].

For implant-associated infection, the location and intensity of these reactions are equally critical. Studies on piezoTi and surface-confined piezocatalysis have shown that when reactive species are generated within the confined space between the implant and bacteria, their utilization efficiency can be markedly improved. Given the extremely short lifetime of ROS, therapeutic efficacy is often determined by whether a sufficiently strong, transient, and spatially confined oxidative stress can be established at the local interface [42,67].

Recent studies have also shifted the focus from single ROS generation to cascade-amplified oxidative and nitrosative reactions. In the BaTiO_3−x_/LA system, ROS trigger NO release, which then reacts with ·O_2_^−^ to form ONOO^−^, thereby markedly enhancing the killing efficiency against MRSA [54]. The BFBT system further couples piezo-sonocatalysis with Fe-based chemodynamic reactions. By promoting Fe(III)/Fe(II) cycling and H_2_O_2_ generation, it achieves more sustained ·OH production and proposes a bacterial ferroptosis-like damage pathway [44].

However, the current antibacterial evidence still has limitations. Most studies use *S. aureus*, MRSA, or *E. coli* as model bacteria and perform validation on flat Ti sheets or in long-bone implant models. These models are useful for mechanistic analysis, but they cannot fully reproduce the salivary acquired pellicle, anaerobic multispecies biofilms, the implant–abutment microgap, or the transmucosal exposure environment. The study by Pan et al., which included oral-related bacteria and used a dental implant model, represents an important step toward clinically relevant oral scenarios [10,11,43,92]. However, the overall evidence remains insufficient for direct extrapolation to the clinical setting of peri-implantitis.

### 4.2. Osteogenesis and Osseointegration

Piezoelectric surfaces promote osseointegration through multiple pathways. First, local electrical signals can alter membrane potential and Ca^2+^ flux, thereby activating osteogenesis-related pathways such as MAPK/ERK, PI3K-AKT, and Wnt/β-catenin. Second, micro- and nanoscale architectures, including nanorods, nanowires, and nanotubes, can regulate protein adsorption, cytoskeletal tension, and focal adhesion maturation. In addition, the release of ions such as Sr^2+^ or Ca^2+^ can further promote osteogenesis through chemical cues at the interface [18,30,31].

Therefore, the optimal piezoelectric surfaces do not always possess the highest coefficients. Instead, they can effectively integrate electrical output, nanotopography, and ionic signaling. Al-STNT combines defect-enhanced ROS/electrical responses with the bone-metabolism-regulating effects of Sr^2+^ [43]. CT-P integrates baseline electroactivity with Ca-related interfacial regulation on the same surface [59]. BST enhances mineral deposition under low-energy stimulation through structural design [41]. These studies suggest that the evaluation of implant surfaces should shift from competition based on a single material parameter to the assessment of integrated interfacial functional synergy.

In vivo studies generally support this trend. PiezoTi restored osseointegration after infection control under infected conditions [42]. BaTiO_3−x_/LA increased new bone volume fraction after MRSA clearance [54]. Al-STNT improved both plaque control and osseointegration in a dental implant model [43]. CT-P and TiO_2_/Bi_2_WO_6_ also demonstrated the potential to promote bone repair under different activation windows [59,61]. However, most existing studies are still short-term experiments in small animals. Their evaluation endpoints and statistical criteria vary considerably, making it difficult to draw truly comparable quantitative conclusions across studies.

### 4.3. Osteoimmunomodulation and Temporally Programmed Repair

After implantation, the local biological outcome is often determined first by immune cells. Macrophages play a central role in inflammation resolution, angiogenesis, and bone repair. If an implant surface is designed only to enhance osteoblast adhesion while neglecting immune regulation, long-term stable osseointegration is often difficult to achieve [13,32,33,34,35].

Recent studies suggest that there is no single optimal macrophage phenotype for all stages of healing. In repair scenarios without infection, or after infection has been controlled, suppressing excessive M1-type inflammation and promoting M2-associated repair responses appears to be more beneficial. This pattern has been clearly observed on TA-Sr and CT-P surfaces [59,70]. However, at infected interfaces, moderate and transient M1-like activation may be necessary, as it can enhance phagocytosis and pathogen clearance. Both piezoTi and BaTiO_3−x_/LA suggest that a stronger pro-inflammatory antibacterial response can be induced during the ultrasound-activated infection clearance stage, while a subsequent shift toward M2-dominated repair is still permitted [42,54].

For dental implants, this stage-dependent regulation is particularly important. If a surface is overly biased toward regeneration during the early contaminated phase, it may fail to eliminate the initial microbial challenge effectively. Conversely, if high oxidative stress or a strong pro-inflammatory state is maintained for too long, host cells may be damaged and osseointegration may be delayed. Therefore, temporal controllability—whether achieved through natural decay of surface potential, on-demand ultrasound switching, or self-limiting cascade reactions—should be regarded as a central design principle for piezoelectric implant surfaces.

### 4.4. Soft Tissue Sealing and Angiogenesis

Unlike general orthopedic implants, dental implants penetrate the oral mucosa and remain continuously exposed to saliva and complex microbial communities. Therefore, osseointegration is not the only endpoint. The quality of soft tissue sealing is also critical, as it determines whether contamination can spread apically along the cervical region of the implant. Recent studies on barrier-erecting implant surfaces have shown that surface wettability, nanotopography, charge status, and collagen- or polyphenol-based molecular layers may influence epithelial adhesion, fibroblast organization, and collagen orientation. These factors can further affect the stability of cervical sealing [70,82,93,94,95,96,97,98,99,100].

For piezoelectric surfaces, direct evidence regarding soft-tissue sealing around dental implants remains limited. Most current studies still focus on osseointegration, antibacterial activity, or osteoimmune regulation as the main outcomes. However, evidence from related electroactive and piezoelectric wound-healing systems suggests that local electrical signals may benefit soft-tissue cells. For example, ultrasound-activated biodegradable piezoelectric PLLA scaffolds have been shown to promote the proliferation of fibroblasts and epithelial cells. They also increased the expression of wound-healing-related genes, such as collagen I, collagen III, and fibronectin, while inhibiting bacterial growth [101]. Electrospun P(VDF-TrFE) piezoelectric nanofibers have also enhanced fibroblast proliferation through mechanically generated surface charges s [102]. Although these studies were not based on dental implant coatings, they support the possible role of piezoelectric outputs in regulating cells involved in soft-tissue repair.

In studies on dental implant surfaces, more direct evidence currently comes mainly from conventional or non-piezoelectric mucosal surface designs. For example, nanotubular titanium surfaces have been reported to enhance the adhesion and migration of human gingival fibroblasts compared with untreated titanium. Titanium surfaces modified with bioactive peptides or calcium have also improved epithelial or connective tissue attachment [103,104,105]. These findings indicate that soft-tissue sealing is strongly affected by nanoscale surface topography, hydrophilicity, surface charge, and bioactive molecular cues [106]. Therefore, piezoelectric implant surfaces should not be evaluated only by osteogenic markers. Future studies should directly compare piezoelectric coatings with machined, anodized, SLA, or hydrophilic titanium surfaces using human gingival fibroblasts and gingival epithelial cells. Key outcomes should include early cell adhesion, proliferation, migration, F-actin organization, collagen I/III deposition, laminin-332 and integrin α6β4 expression, hemidesmosome-related markers, mucosal attachment strength, and resistance to bacterial microleakage at the mucosal interface [70,82,100,107,108,109].

Therefore, the potential role of piezoelectric surfaces in soft-tissue sealing should currently be regarded as promising but not yet fully validated. The next stage should move beyond indirect inference based on osteogenic or antibacterial outcomes. It should focus on direct testing of fibroblast organization, epithelial sealing, collagen orientation, and transmucosal barrier stability under oral-like bacterial and mechanical stresses.

### 4.5. Temporally Programmed Control from Antibacterial Activity to Osteogenesis

The true competitiveness of piezoelectric implant surfaces may not lie in their instantaneous output intensity. Instead, it may depend more on whether they can achieve task switching. From a clinical perspective, the early stage after implantation mainly requires contamination control, reduced biofilm adhesion, and preservation of host defense. Once infection is controlled, the main task of the surface shifts toward promoting inflammation resolution, vascularization, and osseointegration (Figure 3). PiezoTi, BaTiO_3−x_/LA, CT-P, TiO_2_/Bi_2_WO_6_, and SrTiO_3_ nanocannons have demonstrated this concept from different perspectives [42,54,59,61]. Through ultrasound switching, polarization decay, self-limited cascade reactions, or on-demand drug release, surface functions can be shifted from a highly reactive antibacterial state to a low-reactivity pro-regenerative state [58,64,77,78,93].

**Figure 4:**
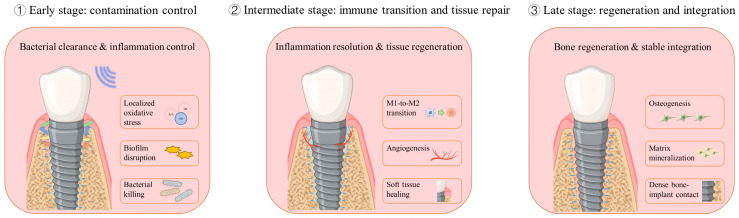
Stage-resolved healing functions of piezoelectric implant surfaces.

Figure 4 illustrates a stage-dependent strategy for piezoelectric and electroactive dental implant surfaces. In the early stage, the main goal is infection control. Local oxidative stress, biofilm disruption, and direct bacterial killing are needed to reduce the bacterial burden and prevent early peri-implant infection. Strong reactive outputs, such as ROS/RNS generation or on-demand antibacterial release, may therefore be beneficial, as long as they remain safe for host tissues.

In the intermediate stage, the biological priority shifts from bacterial elimination to immune transition and tissue repair. A timely shift from an inflammatory M1-dominant response to a reparative M2-associated microenvironment is important for inflammation resolution. Meanwhile, angiogenesis and soft-tissue healing help rebuild the peri-implant barrier and create a supportive microenvironment for later bone regeneration.

In the late stage, the main objective is regeneration and stable integration. Mild and sustained electrical cues, ionic signals, and osteogenic microenvironmental regulation can promote osteogenesis, extracellular matrix mineralization, and dense bone–implant contact. Therefore, the main value of stage-dependent piezoelectric surfaces is not to maintain constant high-intensity antibacterial activity, but to match surface functions with the biological needs of each healing stage.

## 5. Emerging Enhancement Strategies and Design Principles

### 5.1. Heterojunction and Co-Catalytic Strategies

Heterojunction design is one of the most important approaches for converting limited piezoelectric output into effective biological outcomes. In the Au/BTO system, a Schottky interface is formed to prolong electron lifetime [42]. TiO_2_/Bi_2_WO_6_ relies on band alignment and oxygen vacancies to enhance charge carrier separation and NIR responsiveness [61]. TiO_2_-SnO_2_-RuO_2_ further combines multilayer heterojunction transport with pseudocapacitive energy storage [62].

These studies suggest that piezoelectricity alone is not sufficient. To address peri-implant infection and impaired osseointegration, piezoelectric matrices should be integrated with problem-oriented functional components. For infection control, Au cocatalysts, TiO_2_/Bi_2_WO_6_ heterojunctions, oxygen vacancies, Fe-containing redox centers, Al-doped SrTiO_3_/TiO_2_ interfaces, and L-arginine-derived NO can enhance charge separation, ROS/RNS generation, and radical-chain antibacterial reactions [42,43,44,54,61]. For bone repair and immune remodeling, Sr^2+^-releasing SrTiO_3_, CaTiO_3_-based coatings, and Sr-containing metal–phenolic networks can provide osteogenic and immunomodulatory ionic cues [59,60,70]. Drug-delivery components, such as antibiotic-loaded SrTiO_3_ nanotubes combined with NH_4_HCO_3_ gas-generation systems, may further enable on-demand antibacterial release and sustained enhancement of osseointegration [53]. Thus, therapeutic performance depends on the coordinated coupling of piezoelectricity, catalytic chemistry, ion release, drug delivery, and immune–osteogenic regulation.

### 5.2. Doping and Defect Engineering

Al doping, oxygen vacancies, and multielement solid solutions have become some of the most active enhancement strategies in recent years. Al-doped SrTiO_3_/TiO_2_ nanotubes improve ROS generation under ultrasonic stimulation through defect states [43]. BaTiO_3−x_ uses oxygen vacancies to couple with the NO cascade reaction [54]. BFBT further integrates oxygen vacancies with the redox activity of Fe sites [44]. However, defect engineering does not follow a simple “more is better” rule. Excessive oxygen vacancies may increase leakage current, weaken polarization stability, or impair cytocompatibility. Future studies should report not only the catalytic benefits of defect introduction, but also the accompanying changes in piezoelectric performance, surface potential retention, and possible biological costs.

### 5.3. Cascade Reactions and Multimodal Synergy

A clear trend in current multifunctional surface design is the expansion of single-step ROS-mediated antibacterial activity into programmable reaction networks. Examples include the ROS–NO–ONOO^−^ cascade amplification in BaTiO_3−x_/LA [54], the sonodynamic–chemodynamic coupling in BFBT [44], the on-demand drug release and sustained Sr^2+^ release strategy in SrTiO_3_ nanocannons [60], and the multi-window design of TiO_2_/Bi_2_WO_6_, which enables antibacterial action first and then cell mechanics-driven osteogenesis [61]. These studies indicate that advanced implant surface research is shifting from making a single material more active toward designing switchable interfacial reaction programs. What truly matters for clinical translation is not the number of combined modes, but whether these modes can match the clinical sequence of contamination clearance, inflammation resolution, and bone repair.

### 5.4. Design Principles for Implant Translation

For implant translation, several design principles are emerging from the current literature. Electrical outputs should be measured under the same mechanical or ultrasonic conditions used in biological experiments, rather than inferred only from PFM images or theoretical parameters. Antibacterial design should prioritize interfacial confinement and spatial precision, because a higher total radical yield in bulk fluid does not necessarily translate into better local efficacy. In addition, infection control and tissue repair should be treated as stage-specific tasks. A single surface is unlikely to perform optimally in all biological phases unless its outputs can be switched or self-limited. Finally, manufacturing reproducibility, sterilization compatibility, and long-term service stability should be evaluated as core translational parameters.

### 5.5. Standardized Evaluation Checklist

Unlike conventional studies on surface roughening, piezoelectric surfaces involve at least three lines of evidence: materials science, physics, and biology. Reporting only X-ray diffraction, X-ray photoelectron spectroscopy, and PFM results is insufficient to explain in vivo efficacy. Likewise, reporting only reduced colony-forming unit counts or increased alkaline phosphatase activity is not enough to prove a piezoelectric mechanism. For translation into dental implant applications, the basic reporting unit should follow a structure–output–reaction–outcome logic. At the structural level, phase composition, defects or doping, polarization state, and coating adhesion should be reported. At the output level, dynamic electrical signals under realistic stimulation conditions, together with ROS/RNS evidence, should be provided. At the reaction level, bacteria, biofilms, and host cells should be evaluated simultaneously. At the outcome level, attention should be given to osseointegration, soft tissue sealing, and long-term stability [27,44,57,58,64,77,78,110].

In addition, standardization for oral applications should consider the effects of the salivary acquired pellicle, multispecies anaerobic biofilms, osseointegration, soft tissue interfaces, and common cleaning or sterilization procedures on surface performance. It is also recommended that threaded dental implant geometry, occlusal loading, and the parameters of portable ultrasonic devices be incorporated into experimental design. Only under conditions that more closely reflect clinical reality can the true advantages and limitations of piezoelectric surfaces be accurately identified [11,45,82,100,111,112,113] (Table 4).

## 6. Translational Barriers and Future Directions 

### 6.1. Mechanistic Attribution and Evaluation Standards

One of the most prominent current challenges is the lack of rigorous mechanistic attribution. In many studies, excellent therapeutic effects observed under ultrasonic stimulation are directly attributed to the piezoelectric effect. However, depolarized samples, non-piezoelectric chemical analogues, temperature monitoring, or cavitation controls are often not included. Similarly, electrical characterization sometimes remains limited to PFM or surface potential mapping, without output voltage or current data corresponding to realistic ultrasonic parameters or clinically relevant loading conditions [25,26,27,28,45,114]. If this gap remains unresolved, the true contribution of piezoelectric surfaces may continue to be either overestimated or underestimated.

A minimum evidence unit should therefore include mechanical or ultrasonic parameters, electrical outputs, reactive species profiles, temperature and cavitation monitoring, and parallel evaluation of bacterial and host–cell responses. Only when the materials science, physical, and biological evidence are all complete can the proposed piezoelectric mechanism become truly comparable across studies (Table 5).

### 6.2. Stability Under Oral Conditions

Dental implants are threaded three-dimensional devices that must withstand multiple challenges, including insertion torque, cyclic occlusal loading, salivary protein coating, microbial colonization, and clinical cleaning or sterilization procedures. Although current studies have demonstrated good short-term interfacial bonding at the materials level, evidence remains limited regarding polarization retention, adhesion after fatigue, wear debris, changes in phase composition, and the influence of the salivary acquired pellicle on piezoelectric output and ROS diffusion [1,2,60,67].

First, coating fatigue during implant placement is a key issue. During placement, the coating is exposed to insertion torque, friction against cortical and trabecular bone, shear stress at thread edges, and compression in the apical and coronal regions. These stresses may cause microcracks, delamination, local wear, exposure of the titanium substrate, or loss of active piezoelectric domains. This issue is particularly important for brittle ceramic coatings, such as barium titanate, strontium titanate, calcium titanate, and bismuth-based coatings [84,115].

Second, sterilization compatibility should be regarded as a core translational parameter. Autoclaving, gamma irradiation, ethylene oxide treatment, and low-temperature plasma treatment may affect residual polarization, defect states, dopant valence states, surface hydration, polymer chains, ion release, and coating adhesion. Therefore, sterilization should be included as a before-and-after variable [59,66,116,117]. It should be assessed by comparing PFM amplitude and phase, surface potential, voltage/current output, ROS/RNS generation, ion release, and antibacterial or osteogenic performance.

Third, the geometry of threaded implants creates additional challenges. Uniform coating thickness, crystal orientation, defect distribution, and polarization may be difficult to achieve in thread valleys, sharp edges, root regions, and internal microgrooves. Therefore, coatings that perform well on polished disks may behave differently on threaded implants [72,118].

Fourth, ultrasound accessibility should be considered for oral applications. Although ultrasound penetrates tissue better than light, the posterior oral region is affected by limited mouth opening, cheek thickness, tongue position, saliva, prosthetic structures, and probe angle. These factors may reduce coupling efficiency and cause the acoustic dose at the implant surface to differ from the nominal device output. Miniaturized intraoral probes or customized coupling attachments may be needed to achieve reproducible activation in chairside or home-use settings [119,120].

Finally, long-term performance should be evaluated under oral-like conditions. These conditions should include artificial saliva, pH changes, temperature fluctuations, cyclic loading, repeated ultrasound activation, simulated toothbrushing or professional cleaning, fluoride exposure, and polymicrobial anaerobic biofilm challenge. Only coatings that maintain a stable structure–output–function relationship after these tests can be considered realistic candidates for translation into dental implant applications [12,113,121,122,123].

### 6.3. Biosafety and Dose Window

High antibacterial efficacy does not necessarily indicate high safety. For piezo-sonocatalytic surfaces, ROS/RNS that can damage bacterial membrane structures and metabolic systems may also affect host cell viability and repair quality if the dose or exposure duration is not properly controlled. For systems containing Ba, Bi, W, Fe, or noble metals, long-term ion release, local accumulation, and migration of wear debris also need to be included in safety evaluations in a more systematic manner. This is particularly important when repeated ultrasonic activation may be required clinically. At present, evidence remains insufficient regarding whether long-term, low-dose, repeated stimulation may lead to cumulative biological effects [44,54,59,61,62].

### 6.4. Future Research Priorities

The next step is not simply to expand the material library, but to build standardized evidence chains under realistic oral conditions. Specifically, greater emphasis should be placed on developing multispecies anaerobic biofilm models containing salivary acquired pellicles, macrophage–osteoblast co-culture systems, dental implant animal models that include soft tissue sealing assessment, and surface fabrication processes that better match clinically relevant implant geometries.

In terms of material design, biocompatible ceramic and organic–inorganic composite surfaces remain worthy of further investigation [21,22,28,114,124]. In addition, region-specific surface design will become an important direction. For example, switchable antibacterial activity may be strengthened in the soft tissue or cervical region, whereas osteogenic electro-ionic signaling may be enhanced in the intraosseous segment. Another promising strategy is to integrate piezoelectric surfaces with digital manufacturing, personalized thread geometry, and standardized ultrasonic devices, thereby establishing material–device integrated solutions that are closer to clinical workflows. Finally, multi-omics approaches and spatial immune analyses will help define which intensity and timing of electrical or oxidative stimulation are most favorable for osteoimmune repair. These insights will provide a mechanistic basis for truly precise surface design.

### 6.5. Device–Material Synergy for Clinical Translation

The clinical translation of piezoelectric implants is a typical device–material synergy problem. Their therapeutic effects depend not only on whether the surface can respond to stimulation, but also on whether an appropriate dose of mechanical energy can be stably coupled to the peri-implant region. The work by Pan et al. suggested that portable ultrasound devices may provide a potential route for patient self-management, while the SrTiO_3_ nanocannon system demonstrated the feasibility of device-assisted NIR-triggered on-demand drug release [43,60]. Local oral use will require miniaturized ultrasound probes and reproducibly positioned coupling accessories. Only with such device-level support can material activation become a reproducible clinical workflow [45,56,57,58,82].

Current clinical evidence has not yet been matched by clinical trials of piezoelectric dental implant coatings themselves. Most available evidence comes from related physical stimulation strategies, such as LIPUS, pulsed electromagnetic fields (PEMF), or electrical stimulation. These approaches provide useful translational references for device-assisted piezoelectric surfaces [125,126].

For example, a clinical study of LIPUS after dental implant placement reported potential benefits in preserving marginal bone around implants, promoting soft-tissue healing, controlling pain, and improving oral health-related quality of life [125]. In addition, orthopedic studies suggest that LIPUS and electromagnetic stimulation have been clinically investigated for bone repair, although the strength and scope of the evidence remain variable [126]. These trials should be interpreted as indirect clinical support for the feasibility of non-invasive physical activation, rather than direct evidence for the clinical efficacy of piezoelectric implant coatings.

**Figure 5:**
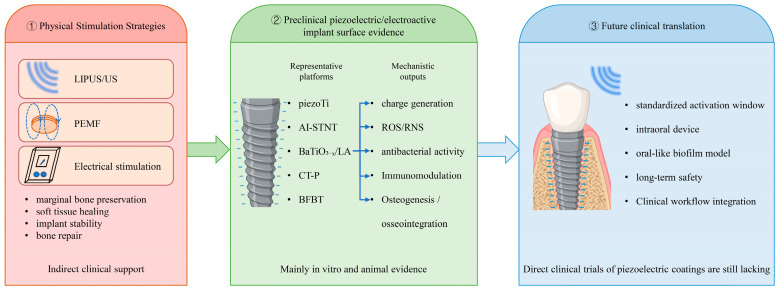
Translational evidence pathway for piezoelectric dental implant surfaces.

At present, piezoelectric or piezocatalytic dental implant coatings are still mainly evaluated through in vitro studies and small-animal preclinical models. Therefore, their translational pathway should be viewed as an indirect evidence chain. Physical stimulation used in clinical practice provides a feasible activation window and device concept. Piezoelectric surfaces tested in preclinical studies provide material-based mechanisms, including charge generation, reactive oxygen/nitrogen species production, immunomodulation, and osteogenesis. Future clinical translation will require standardized material–device systems, oral-like testing models, and long-term safety assessment (Figure 5).

In addition, self-powered smart implants may move piezoelectric surfaces from a state of passively awaiting stimulation toward a new stage of continuous sensing and feedback regulation. Piezoelectric layers can serve as microenergy harvesting units. They may also be integrated with microsensor chips, energy-storage layers, or remote monitoring modules to record local pH, temperature, loading, or infection-related risk signals. Although this direction currently remains largely at the stage of conceptual design or in vitro validation, it deserves to be advanced in parallel with surface biology in future studies [20,56,57,58,63,76,93].

### 6.6. AI/High-Throughput Screening, Multi-Omics, and Personalized Design

The optimization of piezoelectric implant surfaces should not continue to rely on empirical material screening. For high-dimensional design variables such as doping, oxygen vacancies, heterointerfaces, and composite ratios, density functional theory, machine learning, and high-throughput computation are better suited to a “screen first, validate later” strategy. These approaches can help predict band positions, trends in piezoelectric response, defect stability, and potential biosafety windows before material fabrication. In this way, they may shorten the cycle from material discovery to experimental validation [23,64,75,110,127].

At the biological validation level, single-cell transcriptomics, spatial transcriptomics, proteomics, and organoid models are also expected to reshape our understanding of piezoelectric surfaces. These tools can address questions that are difficult to answer using conventional endpoint measurements [20,57,58,64,76,77,78,79,108,110,128]. For example, what intensity and duration of electrical or oxidative stimulation are most favorable for the M1-to-M2 transition? Do piezoelectric signals primarily act on macrophages, osteogenic progenitors, endothelial cells, or fibroblasts? Are the optimal surface parameters the same under different oral disease backgrounds, such as diabetes, smoking, or osteoporosis?

## 7. Conclusions

Piezoelectric surface engineering provides a research pathway for dental implants that differs from conventional passive modification. It integrates mechanical stimulation, local bioelectric signals, interfacial catalysis, and immune remodeling within the same surface. Current studies have shown that BTO, strontium-containing titanates, CaTiO_3_, Bi_2_WO_6_, and multicomponent heterostructures can achieve coordinated antibiofilm and antibacterial activity, osteogenesis and osseointegration, and osteoimmunomodulation in different scenarios. Among these strategies, surface-confined catalysis, temporally programmed switching, and electro-ionic-topographical synergy represent the most promising design paradigms for further development.

Nevertheless, most available evidence still comes from materials studies and small animal preclinical models. Several clinically relevant issues remain insufficiently addressed, including multispecies biofilms, complex oral biomechanics, long-term polarization stability, soft tissue sealing, and engineering manufacturability. The next stage should aim to establish reproducible, scalable, and regulatory-compatible surface systems based on standardized evaluation and realistic oral scenarios. Only by integrating piezoelectric mechanism, oral application models, and engineering feasibility can piezoelectric materials move from a conceptual frontier toward clinically applicable.

## Figures and Tables

**Table 1:** Representative piezoelectric dental implant surface systems and their functional characteristics.

Surface System	Activation Condition	Key Design Features	Quantitative Output/Endpoint	Mechanistic Evidence	Main Limitation	Representative Study
piezoTi/BTO-Au	US, 1 MHz	In situ BTO growth + Au electron sink/co-catalysis	ROS generation, antibiofilm activity, enhanced macrophage antibacterial response	BaTiO_3_ piezoelectricity + Au cocatalyst-mediated charge separation; macrophage phagocytosis activation	Mainly preclinical infection models; oral multispecies biofilm and dental implant geometry still insufficient	Li et al. [42]
BST nanorod arrays	LIPUS, 1.0 MHz, output power 5.0 W; polarized BST prepared at 11.5 kV, 120 °C, 20 min	Co-optimization of Ba/Sr ratio and nanorod-array morphology	No obvious cytotoxicity; improved osteoblast adhesion/proliferation; enhanced mineralization behavior in SBF	Rod-array microstructure improved response to small mechanical/ultrasonic inputs; PFM confirmed piezoelectric properties; simulated and experimentally verified positive/negative surface potential distribution	Mainly in vitro mineralization and osteoblast assays; antibacterial, oral biofilm, threaded implant, and long-term fatigue data are limited	Wu et al. [41]
BTO_−x_/LA nanorod arrays	US, 1 MHz, 1.5 W/cm^2^, 50% duty cycle, 15 min	Oxygen vacancies + L-arginine grafting	In vivo antibacterial rate 97.54%; BV 0.61 mm^3^ and BV/TV 34.26% at 8 weeks	Oxygen vacancies; ROS–NO–ONOO^−^ radical chain reaction; early M1 and later M2-stage regulation	Tibial model; oral biofilm and threaded dental implant conditions not fully reproduced	Sun et al. [54]
Al-SrTiO_3_/TiO_2_ nanotubes	US, 1 MHz, 1.5 W/cm^2^, 50% duty cycle, 5 min	Anodized template + Al-doping-induced oxygen vacancies	Killing rates of *P. gingivalis* and *F. nucleatum*: 80.4% and 82.1%; Sr^2+^ release about 9.18 μg/mL by day 7	Al doping, oxygen vacancies, SrTiO_3_/TiO_2_ heterojunction, enhanced ROS generation	Repeated activation, oral aging, and long-term coating stability require more testing	Pan et al. [43]
BFBT defect-enriched nanoreactor	US-triggered activation; typical antibacterial tests used 1 MHz, 1.5 W/cm^2^, 50% duty cycle, 5 min	Fe sites + oxygen vacancies + cascade catalysis	Strong in vitro and in vivo antibacterial effects; enhanced bone regeneration and macrophage immunomodulation	Oxygen-vacancy-rich BFBT; US-driven built-in electric field; self-supplied H_2_O_2_; Fe(III)/Fe(II) cycling; SPT–CDT tandem catalysis; iron-metabolism disruption and ferroptosis-like bacterial death	Mechanism is highly complex; difficult to separate piezoelectric, chemodynamic, Fe-metabolism, HA ion-release, and scaffold/topography contributions	Zheng et al. [44]
Polarized CaTiO_3_ (CT-P)	Baseline electrical activity + US, 1 MHz, 1.5 W/cm^2^, 10 min	Polarization-derived time-dependent surface potential	RhB degradation >50% after 12 h under US; antibacterial and osteogenic effects reported	Time-dependent surface potential; US-triggered ROS; immune and osteogenic regulation	Polarization stability after sterilization and insertion remains unclear	Dai et al. [59]
TiO_2_/Bi_2_WO_6_ heterojunction	NIR for antibacterial activation; cell traction for osteogenic electrical cues	Built-in electric field + oxygen vacancies + heterogeneous interface	Antibacterial and osteogenic outputs reported	Heterojunction charge transfer; oxygen-vacancy-induced intermediate band; light-cellular force-electric coupling	Not a purely piezoelectric antibacterial system; photothermal/photodynamic effects overlap	Fan et al. [61]
TiO_2_-SnO_2_-RuO_2_ electroresponsive surface	Post-charging/endogenous electrical stimulation	Multilayer heterojunction + pseudocapacitive energy storage	Improved antibacterial activity and osseointegration after negative post-charging	Built-in electric field, pseudocapacitive charge storage, surface redox regulation	Requires standardized charging protocol and charge-retention evaluation	Zhou et al. [62]

**Table 2:** Main construction routes and processing considerations for piezoelectric implant surfaces.

Route	Typical Procedure	Advantages	Disadvantages	Representative Systems
Hydrothermal in situ growth	Alkaline hydrothermal pretreatment to form a titanate precursor layer, followed by hydrothermal conversion into BTO/BST/CT nanostructures	Relatively strong interfacial bonding; controllable morphology; certain conformality on complex surfaces	Subsequent heat treatment may introduce brittleness, residual stress, and microcracks; phase formation and polarization state may be difficult to control or maintain during long-term service	BTO/Au, BST, CT-P
Anodization-hydrothermal coupling	Preparation of ordered TiO_2_ nanotubes, followed by in situ formation of SrTiO_3_ or subsequent loading/doping	Nanotube templates facilitate drug storage and transport, ion release, and stress transfer	Multiple processing steps increase batch-to-batch variation; nanotube wall collapse, incomplete phase transformation, and local thickness heterogeneity may occur	Al-STNT, SrTiO_3_ nanocannon
Poling treatment	Corona poling or high-voltage poling to increase surface potential and charge-retention capacity	Markedly enhances baseline electroactivity and introduces temporally programmed functions	Depolarization after sterilization, wet aging, insertion torque, and cyclic oral loading remains a major risk; standardized clinical poling/charging protocols are lacking	CT-P, BST
Doping/defect engineering	Introduction of Al, oxygen vacancies, or multielement solid solutions to regulate band structure and polarization behavior	Enhances charge carrier separation and ROS yield; may enable new reaction pathways	Excessive defects may increase leakage current, reduce polarization stability, alter ion-release behavior, complicate mechanism attribution, and compromise cytocompatibility	BTO_−x_/LA, Al-STNT, BFBT
Heterojunction/co-catalytic layers	Au, Bi_2_WO_6_, RuO_2_, and related components form interfacial synergy with the main piezoelectric layer	Improves charge utilization efficiency and expands multimodal functions	Complex structure, higher cost, and possible interfacial instability; difficult to distinguish piezoelectric effects from photothermal, photodynamic, pseudocapacitive, ionic, or topographical effects	piezoTi, TiO_2_/Bi_2_WO_6_, TiO_2_-SnO_2_-RuO_2_
Polymeric/composite piezoelectric systems	Electrospinning, casting, stretching, poling, or inorganic filler incorporation to form flexible piezoelectric films, membranes, or scaffolds	Flexible, processable, and suitable for soft-tissue interfaces, barrier membranes, or local drug delivery	Lower mechanical durability and weaker adhesion on threaded metallic implants; piezoelectric output depends strongly on molecular orientation and polarization; sterilization and long-term wear resistance remain uncertain	PVDF-based composites, PLA/PLLA systems

**Table 3:** Critical comparison of representative piezoelectric and electroactive implant surface strategies.

Strategy	Typical Activation Condition	Dominant Mechanism	Advantages	Limitations
Endogenous-force-responsive piezoelectric surfaces	Mastication, cell traction, or local micromotion	Mechano-electrical conversion and local electrical stimulation	No external device required; suitable for long-term osteogenic or immunomodulatory cues	Output intensity may be weak and heterogeneous; difficult to quantify under real oral loading
Ultrasound-activated piezodynamic surfaces	LIPUS or US	Piezoelectric charge separation and ROS/RNS generation	Noninvasive activation; strong antibacterial and antibiofilm potential	Efficacy depends on ultrasound attenuation, probe angle, coupling medium, tissue thickness, and safety window
Defect-/doping-enhanced piezo-sonocatalytic coatings	US combined with oxygen vacancies or aliovalent doping	Enhanced charge separation, increased ROS/RNS yield, and improved redox reactions	Higher catalytic efficiency; useful for infection control	Defects and dopants may complicate biosafety, stability, and mechanism attribution
Heterojunction/cocatalyst-based systems	US, light, or cell traction depending on design	Built-in electric field, carrier separation, and interfacial redox reactions	Integrates antibacterial activity and bone regeneration; can reduce charge recombination	Multiple effects may overlap, including photothermal, photodynamic, topographical, and piezoelectric mechanisms
Electroresponsive pseudocapacitive surfaces	Pre-charging or post-charging protocols	Charge storage, surface redox-state regulation, and electrical stimulation	Provides controllable surface charge and electrical output; useful for antibacterial and osteogenic regulation	Requires standardized charging conditions; long-term charge retention and clinical workflow remain unclear
Ion-/drug-release synergistic platforms	NIR, US, pH, or time-dependent release	Controlled antibacterial agent release, ionic signaling, and osteogenic stimulation	Allows temporally programmed antibacterial and regenerative effects	Release kinetics, burst release, repeated activation, and manufacturing reproducibility need careful evaluation
Immune-first synergistic surfaces	Usually no external activation; driven by surface chemistry	Early macrophage modulation, BMSC recruitment, and osteoimmune remodeling	Provides a reference for stage-specific healing design	Not necessarily piezoelectric; should be discussed as a complementary design principle rather than direct piezoelectric evidence

**Table 4:** Recommended evidence chain and minimum evaluation requirements for clinical translation.

Evidence Level	Minimum Evaluation Requirements	Recommended Enhancements
Material structure	Phase composition, crystal orientation, defects/doping, polarization state, coating thickness, and adhesion	Post-fatigue structure; structural stability before and after sterilization; consistency on complex 3D surfaces
Dynamic physical output	Open-circuit voltage/current or surface potential changes under conditions consistent with biological experiments	Simultaneous monitoring of temperature rise, cavitation, and local acoustic field
Catalytic/chemical reactions	Types and quantitative evidence of ROS/RNS, verified by at least one scavenger or control	Evidence for interfacially confined reactions; time-resolved reaction kinetics
Antibacterial evaluation	Plate counting + biofilm imaging + at least one membrane damage or metabolic indicator	Salivary acquired pellicle; multispecies anaerobic biofilm; recolonization model
Host response	Macrophage polarization, osteogenic differentiation, and cytocompatibility	Fibroblast/epithelial/endothelial co-culture; spatial omics or transcriptomics
Animal model	Implant stability, new bone volume, and inflammation assessment	Dental implant geometry; transmucosal interface; occlusal loading; large-animal oral model
Device-enabled pathway	Stimulation parameters, probe position, coupling medium, and frequency of use	Portable devices, personalized therapeutic windows, and follow-up management programs

**Table 5:** Key gaps and suggested evaluation indicators for translational research on piezoelectric dental implants.

Key Gap	Nature of the Issue	Suggested Evaluation Indicators/Experimental Considerations
Insufficient mechanistic attribution	Piezoelectric effects, acoustic cavitation, local temperature rise, and general sonochemical effects should be analyzed comprehensively	Include depolarized and non-piezoelectric controls; monitor temperature and cavitation; report electrical output and ROS/RNS evidence in parallel
Disconnect between electrical characterization and biology	PFM or static surface potential is insufficient to represent the real service state	Report open-circuit voltage, current, or surface potential changes under mechanical/ultrasonic conditions consistent with the biological experiments
Limited realism of oral scenarios	Flat specimens and single-species bacterial models are difficult to extrapolate to peri-implantitis	Introduce salivary acquired pellicles, multispecies anaerobic biofilms, dental implant geometries, and soft tissue interface evaluation
Lack of long-term stability evidence	Coating fatigue during insertion, sterilization-induced depolarization, nonuniform coating on threaded geometries, limited posterior ultrasound accessibility, and oral-environment aging are rarely evaluated	Use screw-shaped implants; test coating integrity after insertion torque; compare piezoelectric outputs before and after sterilization; evaluate coating continuity on thread valleys and apical regions; map local acoustic fields for anterior/posterior implants; perform saliva aging, pH cycling, thermal cycling, cyclic loading, repeated ultrasound activation, and post-aging biological assays
Undefined safety window	Antibacterial dose may conflict with host compatibility	Define ultrasonic parameters, safety thresholds, and histological and organ safety after long-term repeated stimulation
Incomplete preclinical evidence chain	Small-animal long-bone models differ substantially from real dental implantation	Include large-animal oral models, standardized follow-up time points, and reproducible device/material combination protocols

## Data Availability

Not applicable.

## Abbreviations

The following abbreviations are used in this manuscript:

US	ultrasonic
ROS	reactive oxygen species
RNS	reactive nitrogen species
LIPUS	low-intensity pulsed ultrasound
BTO	BaTiO_3_
CT	CaTiO_3_
CT-P	polarized CaTiO_3_ coating
NIR	near-infrared
MRSA	methicillin-resistant Staphylococcus aureus
BFBT	(BiFe)_0.9_(BaTi)_0.1_O_3−x_
BV/TV	bone volume fraction
PEMF	pulsed electromagnetic fields
BST	barium strontium titanate
BMSCs	bone marrow mesenchymal stem cells
Ca^2+^	calcium ions
Al-STNT	Al-SrTiO_3_/TiO_2_ nanotubes
PFM	piezoresponse force microscopy
SrTiO_3_	strontium titanate
PDA	polydopamine
PVDF	Polyvinylidene fluoride
P(VDF-TrFE)	Poly (vinylidene fluoride-co-trifluoroethylene)
AlN	Aluminum Nitride
GaN	Gallium Nitride
TA-Sr	tannic acid–strontium
